# Influence of fixed orthodontic steel retainers on gingival health and recessions of mandibular anterior teeth in an intact periodontium - a randomized, clinical controlled trial

**DOI:** 10.1186/s12903-024-03998-0

**Published:** 2024-02-14

**Authors:** Hari Petsos, Ralf Usherenko, Iulia Dahmer, Peter Eickholz, Stefan Kopp, Babak Sayahpour

**Affiliations:** 1 Department of Periodontology, Center of Dentistry and Oral Medicine (Carolinum), Goethe University Frankfurt, Theodor-Stern-Kai 7, 60596 Frankfurt/Main, Germany; 2 Rossmarkt 22–26, 63739 Aschaffenburg, Germany; 3 Institute of Biostatistics and Mathematical Modeling, Goethe University Frankfurt, Theodor-Stern-Kai 7, 60596 Frankfurt/Main, Germany; 4 Center of Dentistry and Oral Medicine (Carolinum), Goethe University Frankfurt, Theodor-Stern-Kai 7, 60596 Frankfurt/Main, Germany; 5 Department of Orthodontics, Center of Dentistry and Oral Medicine (Carolinum), Goethe University Frankfurt, Theodor-Stern-Kai 7, 60596 Frankfurt/Main, Germany

**Keywords:** Retainer, Gingival health, Gingival recession, Periodontitis, Orthodontic treatment

## Abstract

**Objective:**

Aim of this randomized clinical controlled trial was to evaluate the influence of fixed orthodontic steel retainers on gingival health and recessions of mandibular anterior teeth.

**Materials and methods:**

After end of the orthodontic treatment, patients were randomly assigned into the test (fixed steel retainer) or control group (modified removable vacuum-formed retainer). Periodontal parameters (periodontal probing depth: PPD; recession: REC; bleeding on probing: BOP) as well as plaque and gingival index were assessed on mandibular anterior teeth directly before attaching/handing over the retainer (baseline: BL), 6 and 12 months after orthodontic treatment.

**Results:**

37 patients (test: *n* = 15, mean age: 16.1±4.2 years; control: *n* = 17, mean age: 17.1±5.4 years) completed the study. REC and PPD failed to show significant pairwise differences. The number of patients showing gingival health in the area of the mandibular anterior teeth (test: BL *n* = 10, 6 months *n* = 9, 12 months *n* = 11; control: BL *n* = 10, 6 months *n* = 16, 12 months *n* = 15) revealed a significant difference for the intra-group comparison between BL and 6 months in the control group (*p* = 0.043). The inter-group comparisons failed to show significant differences.

**Conclusion:**

Young orthodontically treated patients with fixed steel retainers show in 73.3% healthy gingival conditions after one year which are comparable to the control group (88.2%). Gingival recessions were in a clinically non-relevant range at any time of the examination.

**Clinical trial number:**

DRKS00016710.

## Introduction

The primary challenges orthodontists must correct are misalignments and/or malocclusions. However, the long-term stability of orthodontic treatment is important. Correct positioning of teeth must be maintained for health, function, and aesthetics. To achieve this, periodontally healthy conditions are required. Studies have shown that orthodontic prolapse or secondary crowding of the anterior teeth occurs in 40–90% of orthodontically treated patients [1]. Furthermore, a recent review reported that, in non-periodontitis patients, orthodontic tooth movement and retention have no significant impact on periodontal outcomes [2]. However, whether these periodontal outcomes remain stable during the maintenance phase is unknown.

The most common clinical retention appliances are removable retainers and fixed canine-to-canine retainers. Removable retainers strongly rely on patient compliance [3, 4]. Therefore, fixed retainers were introduced to minimise relapse risk and provide reliable long-term results [5]. The use of fixed retainers in orthodontic practice has been consistently increasing [6]. It is important that these appliances do not compromise individual biofilm control and gingival health. Compromised gingival health is indicated when the periodontal probing depth (PPD) is ≥ 3 mm, there is bleeding on probing (BOP) at ≥ 10% of sites, and the presence of clinical attachment loss (CAL) [7]. Increasing use of fixed retainers has raised concerns regarding their impact on gingival health [2, 8]. Due to these appliances crossing the interdental space, it is difficult to sufficiently practice interdental hygiene in the canine-to-canine areas [9–11].

The current evidence on the impact of orthodontic retainers on periodontal conditions is inconsistent [9, 12–17]. Previously published studies on this topic agree that fixed retainers promote plaque and calculus accumulation. However, there is a different picture regarding the impact on gingival and periodontal health. Rody et al. states that “*the presence of retainers bonded to all anterior teeth seems to increase plaque accumulation and gingivitis*” [12]. Levin et al. even concluded that “orthodontic treatment and fixed retainers were associated with an increased incidence of gingival recession, increased plaque retention, and increased bleeding on probing” [14]. In contrast, the studies of Pandis et al. and Booth et al. concluded “*no significant difference was found with respect to the plaque and gingival indices and bone level between the two groups*” respectively that “*long-term retention of mandibular incisor alignment is acceptable to most patients and quite compatible with periodontal health*” [15, 17].

However, the risk of developing mandibular labial recessions may increase during the maintenance phase [18]. Several clinical trials have recommended further investigations on this topic to be carried out to further assess periodontal effects of retainers by comparing patients treated with fixed retainers to a control group who received a removable retainer or no retainer [19–24]. Because not rendering patients a retainer is unacceptable for ethical reasons; therefore, minimal retention must be guaranteed [19, 21].

The current randomized clinical controlled trial compared the effect of fixed and modified removable orthodontic retainers (in terms of minimal residual retention) on gingival health and recessions of mandibular anterior teeth 12 months after orthodontic treatment completion. The follow-up period was chosen according to the time management of the underlying master’s thesis. Patients in this trial initially presented as periodontally healthy. Periodontal health was based on the definition by Chapple et al. which is essentially characterized by minimal bleeding on probing (< 10%), no critically increased probing depths (≤ 3 mm) and no clinical loss of attachment [7].

## Materials and methods

### Participants and randomisation

All participants were recruited from the Department of Orthodontics, Johann Wolfgang Goethe-University Frankfurt am Main. All participants were orthodontically pretreated due to malocclusion and/or misalignment using fixed orthodontic appliances. In some cases, this was combined with interproximal reduction. Participants had to meet several inclusion criteria to be included in the study:

- minimal age of twelve years,
- presence of all mandibular anterior teeth from canine to canine,
- completion of orthodontic multi-bracket treatment at the department with the requirement of retention of the mandibular anterior teeth,
- periodontal screening index ≤ 2 [25],
- participation to all study visits (visit 1: screening and randomisation, visit 2/baseline: adhesive attachment of fixed-steel retainer or incorporation of removable retainer, visit 3: 6-month re-examination, visit 4: 12-month re-examination).

Pregnant participants as well as participants suffering from systemic diseases or conditions requiring antibiotic prophylaxis for clinical measurements that trigger transitory bacteraemia.

were excluded from the study. At the end of their orthodontic treatment, participants were randomly assigned to one of two groups: the test group (canine-to-canine fixed-steel retainer) or the control group (modified removable vacuum-formed retainer). A randomisation list was generated to carry out the random assignment (URL: www.random.org) by R.U., participants were enrolled and assigned to interventions by B.S.

The study was approved by the Institutional Review Board for Human Studies of the Medical Faculty, Johann Wolfgang Goethe-University Frankfurt am Main (approval number: 95/19). The study was conducted in accordance with the 1975 Declaration of Helsinki, as revised in 2013, and was registered in the German clinical trial register (Deutsches Register Klinischer Studien; ID: DRKS00016710; date of registration: 05/09/2019). All patients provided written informed consent prior to participating in this study.

### Intervention

**Fig. 1:**
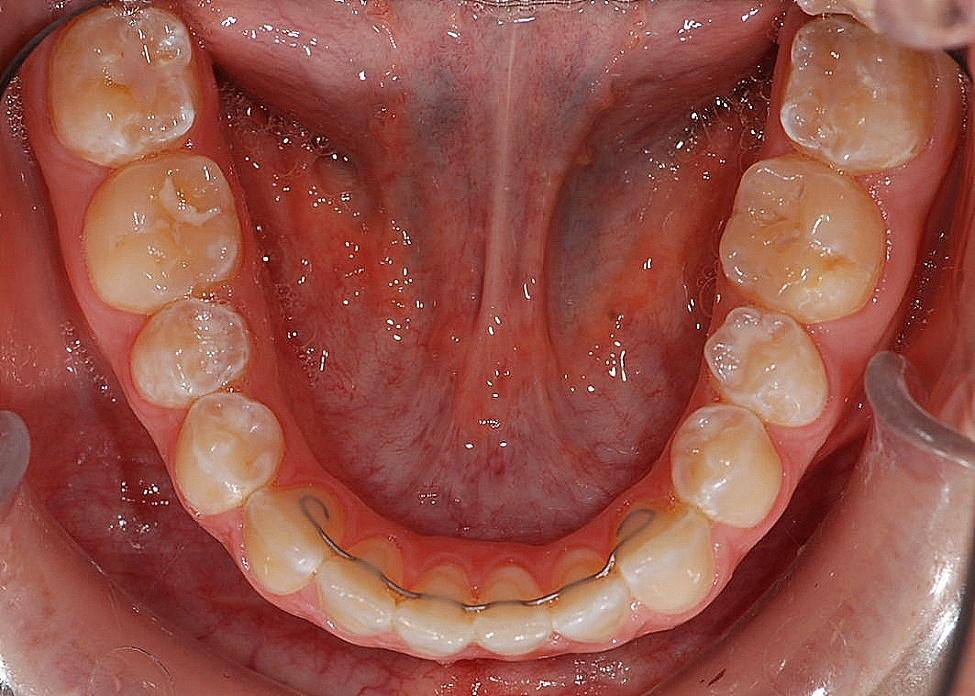
Intraoral view of a lingually bonded fixed steel retainer (test group)

At the end of active orthodontic treatment, brackets were removed using orthodontic pliers. The former adhesive surfaces were treated using finishers (H379 204 023, Gebr. Brasseler GmbH & Co. KG, Lemgo, Germany) and rubber polishers (Brownie Mini-Points, Greenie-Minipoints, SHOFU DENTAL GmbH, Ratingen, Germany). Retainers were inserted on the day of debonding. The fixed retainers for the test group were individually bent on a plaster working cast, fabricated from a stainless-steel alloy (FlexSelect® stainless steel 14”, Flexmedics, Franklin, IN, USA) and applied to each lingual tooth surface from one mandibular canine to the next. Prior to insertion, the retainer was degreased with alcohol. Participants’ teeth were cleaned, polished, preconditioned with 35% phosphoric acid (Ultra Etch®, Ultradent Products Inc., South Jordan, UT, USA), and primed with bonding resin (Ortho Solo™, Ormco, Brea, CA, USA). After this, the wire was positioned and placed freehand into a layer of flowable composite (Tetric Evo Flow A2, Ivoclar Vivadent Schweiz AG, Glattpark, Switzerland). After light curing, any excess composite or bonding resin was removed. Finally, the lingual tooth surfaces were polished using rubber polishers (Brownie- and Greenie-Minipoints, SHOFU DENTAL GmbH, Ratingen, Germany) and fluoridated (Elmex Gelee, CP GABA GmbH, Hamburg, Germany) (Fig. 1) [26].

**Fig. 2:**
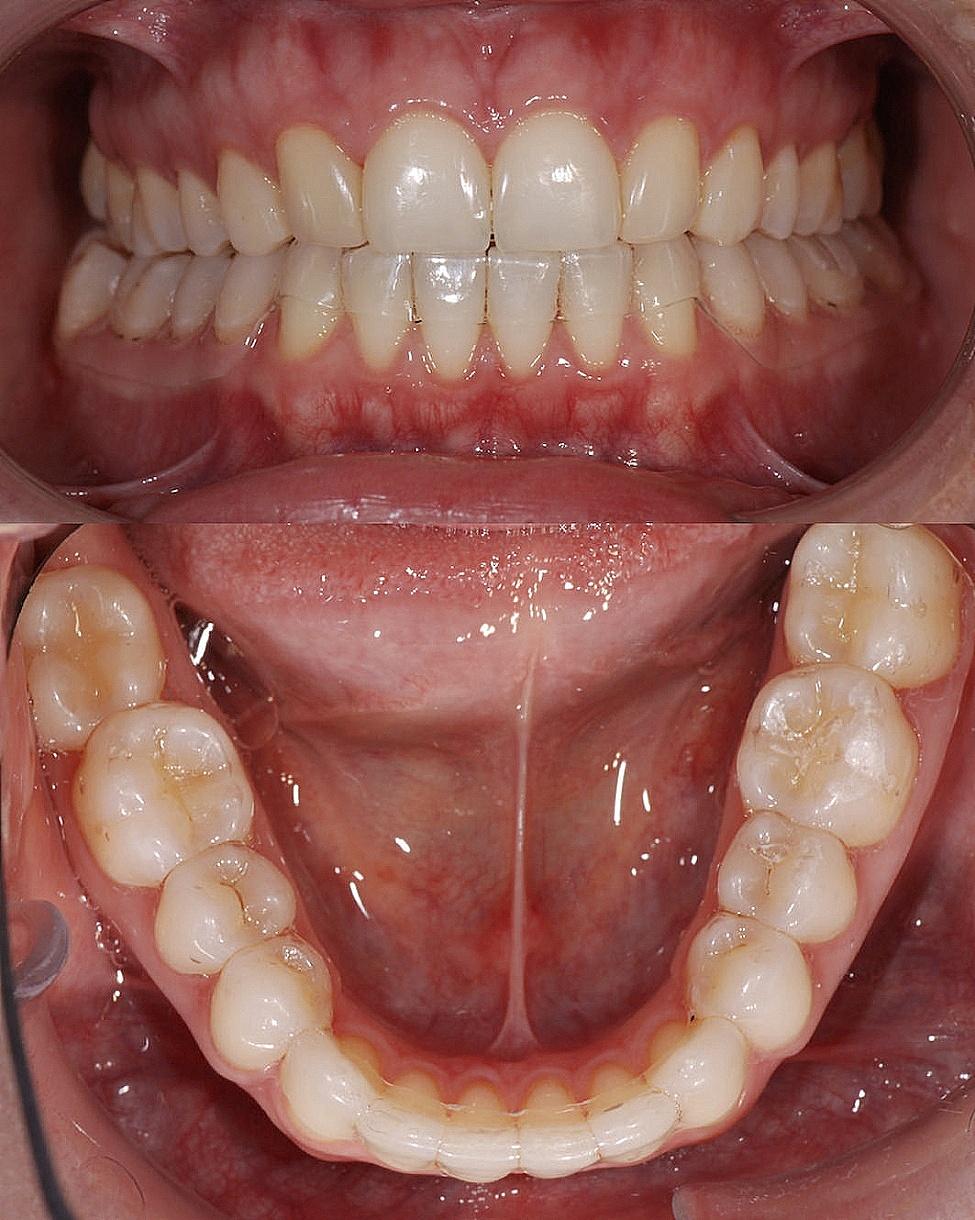
Intraoral view of a modified removable vacuum-formed retainer (control group; upper figure: frontal view, lower figure: occlusal view)

For the control group, the mandibular removable retainers were fabricated using 0.75 mm thick thermoplastic and vacuum-formed plates (Duran®, Scheu-Dental GmbH Iserlohn, Germany). These were further modified by reducing the cervico-incisal height in the intercanine area to minimise the amount of periodontal tissue covered. This was to limit the impact on plaque accumulation and to prevent the development of recessions due to material existing near the gingival margin. An orthodontically treated control group that received no retention would have been unethical since tertiary crowding would be risked. After wearing the retainer for 5 min, the fit and presence of pressure points were checked and adjusted if needed for each participant (Fig. 2).

Upon retainer insertion, oral hygiene instructions were given to all participants. Participants were instructed to use a medium manual (modified Bass cleaning technique) or electric toothbrush with fluoride-containing toothpaste. Additionally, participants were advised to use a fluoride-containing mouthwash solution. Participants in the test group were instructed to use interdental floss. For participants in the control group, it was recommended they clean their removable retainer with water and a soft toothbrush.

In the control group, the minimum retainer wear time was expected to be 22 h a day for the first 3 months, following the retention protocol of the Department of Orthodontics University of Frankfurt, to prevent any potential relapse [27, 28]. After that, participants only had to wear it at night. In addition to the study examinations, all participants received 3-monthly routine check-ups at the Department of Orthodontics to monitor occlusion, oral hygiene, and residual growth. For both groups, retainers were checked and optimized if necessary. In the test group, this involved adhesively re-attaching the trainer. If the removable retainers used by the control group fractured, the retainer was re-manufactured the same day. All retainers were applied by the same orthodontist (B.S.).

### Periodontal parameters

All clinical measurements performed ranged from one mandibular canine to the other and were taken with a rigid, millimeter-scaled periodontal probe (PCP-UNC 15; Hu-Friedy, Chicago, IL, USA) by the same periodontist (H.P.) at baseline (BL) as well as after 6- and 12-month:

#### PPD

PDD was defined as the distance from the gingival margin to the most apical part of the sulcus. It was measured at 6 sites per tooth (mesiobuccal, buccal, distobuccal, mesiolingual, lingual, and distolingual). Measurements were rounded to the nearest 0.5 mm [29].

#### Retainer distance

In the test group, retainer distance was measured on the lingual side of each tooth with a periodontal probe. It was defined as the distance from the retainer to the deepest point of the cemento-enamel junction (CEJ) [14]. Measurements were rounded to the nearest 0.5 mm.

#### BOP

BOP was visually assessed as the proportion of sites that were bleeding 30 s after 6 sites per tooth were probed [29].

#### Gingival recession (REC)

REC was measured from the lowest point of the vestibular and lingual CEJ to the lowest point of the vestibular and lingual marginal gingiva. Measurements were rounded to the nearest 0.5 mm [30].

#### Gingival index (GI) and plaque index (PlI)

The GI and PlI were assessed at 6 sites per tooth, with possible scores of 0–3 given to each site for each index [31]. For further analysis, the GI and PlI scores were dichotomised according to GI/PlI = 0 and GI/PlI ≥ 1 [32].

#### Gingival health (GH)

The criteria set out by Chapple et al. for GH were applied at all time points: BOP < 10%, PPD ≤ 3 mm, and the absence of CAL [7].

### Orthodontic parameters

#### Inclination of the lower incisors before and after treatment

Using cephalometric analysis, the anterior angle was measured between the linear connection of the ‘menton’ (most caudal point of the mandibular symphysis) to the ‘incisura masseterica’ (strongest cranial retraction point in the horizontal mandibular branch) and the ‘incisura inferius” (midpoint on the incisor edge of the most labially positioned mandibular central incisor) to the ‘incisura inferius apicale’ (root tip of the most labial lower central incisor) [33, 34].

#### Interproximal reduction (IPR)

Flexible strips of fine material were used to remove small portions of enamel (IPR System, ContactEZ, Vancouver, WA, USA). This parameter was analysed dichotomously in the analysis [35, 36].

#### Mandibular intercanine distance before and after treatment

The distance between the mandibular canine tips was measured using digital model analysis before and after treatment [37, 38].

#### Dental space deficiency before treatment

Using digital model analysis, the difference between the width of the mandibular anterior teeth and the mandibular intercanine distance was determined. The parameter was analysed dichotomously, where the presence of a space deficiency indicated a negative result [39].

### Statistical analysis

Data were entered in an Excel-based (Excel version 16.23, Microsoft Corporation, Redmond, WA, USA) data matrix. Participants were defined as statistical units. REC was considered the primary outcome measurement. All other study variables were considered secondary outcome parameters. Given a maximum difference of 0.1 mm in REC between the two groups, at least 28 participants were needed to perform a non-inferiority test with a power of 80%, a significance level α of 5%, and a non-inferiority limit Δ = 0.3 mm (assuming a standard deviation of s = 0.2) [14]. The authors chose Δ = 0.3 mm because this was the minimal difference that could be visually identified based on the periodontal probe used in the event of a recession. As there was a possibility that a non-parametric test may be used, and assuming a dropout rate of 20%, in total, 36 participants were recruited.

After testing for normal distribution using a mixed model, where participants were the random effect and study group and visit number were the fixed effects (Shapiro-Wilk test for the residuals, *p* < 0.001), PPD and REC were recorded descriptively using medians, interquartile ranges (IQRs), means, and standard deviations to ensure comparability with other studies. CAL was calculated by adding PPD and REC. As negative RECs were clinically estimated in cases of non-visible CEJ, CAL ≤ 1 mm was accepted as an ‘absence of CAL’. Categorical variables were presented as absolute and relative frequencies.

Differences in REC between the BL and 6-month examinations and between the BL and 12-month examinations were computed and compared between the two groups using a one-sided Mann-Whitney U test with a non-inferiority limit of Δ = 0.3 mm.

Depending on the normal distribution of each parameter, univariate inner-group comparisons of BL, 6 and 12 months numerical parameters were compared using non-parametric Friedman’s 2-way ANOVA (REC) or repeated measures ANOVA (PPD and CAL). Categorial parameters were compared using Cochran’s Q-Test (PlI, GI, BOP, and GH). Inter-group comparisons for the 3 examination time points were performed using the Mann-Whitney U test (REC), t-test (PPD and CAL), or chi-squared or Fisher’s exact tests (PlI, GI, BOP, and GH). A type 1 error of 0.05 was considered statistically significant. *P*-values were adjusted for the inter-group comparisons using Bonferroni correction in order to account for multiple testing. Data were evaluated using the IBM SPSS Statistics 28 software package (SPSS, Chicago, IL, USA).

## Results

### Participants

**Fig. 3:**
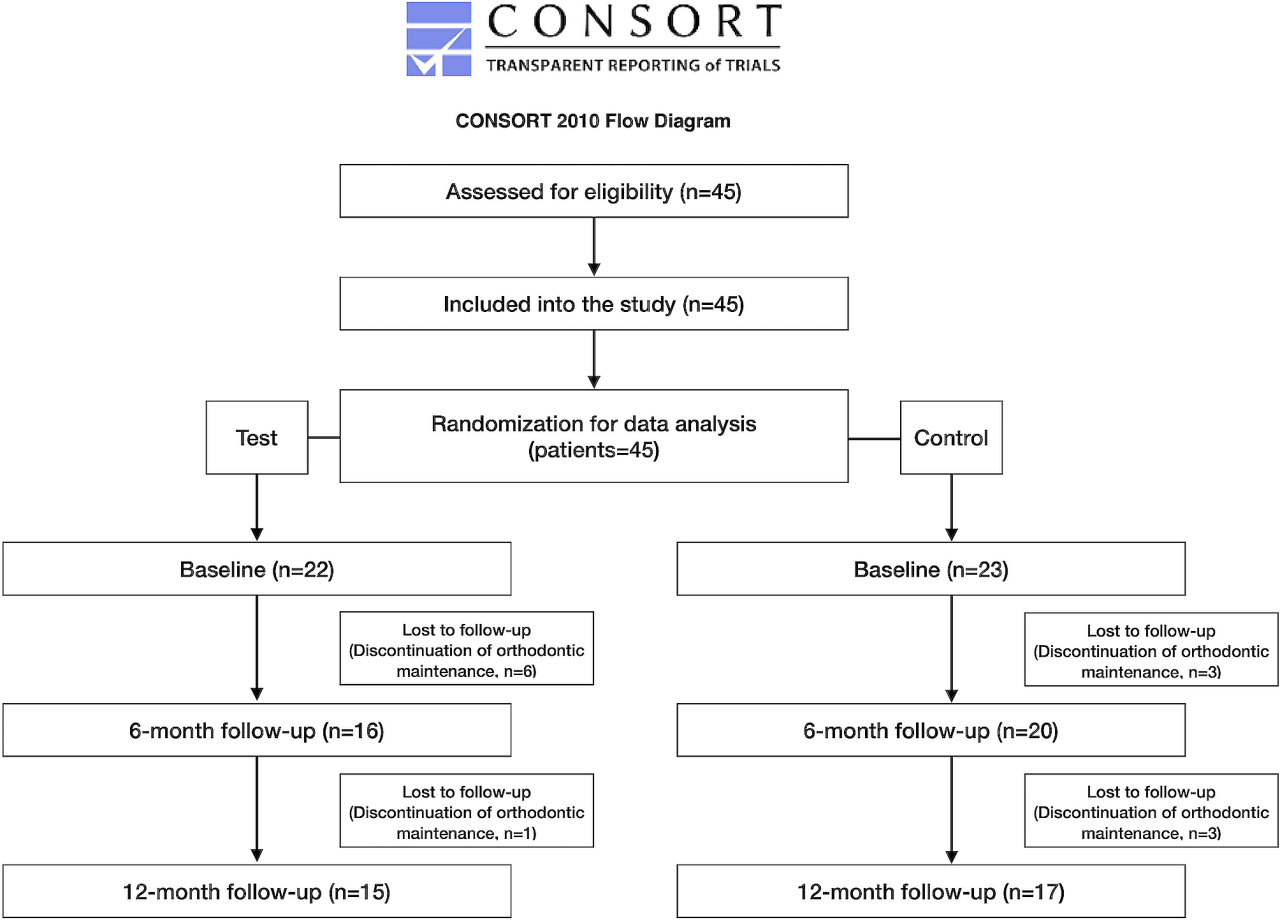
Flow diagram

Of the 45 participants originally included, 32 attended the 12-month examination. Nine patients dropped out between the BL and 6-month examinations, and a further 4 dropped out between 6- and 12-month examinations. All of them discontinued orthodontic maintenance (Fig. 3). The test group comprised 15 participants (11 female, mean age 16.1±4.2 years). The control group comprised 17 participants (11 female, mean age 17.1±5.4 years). None of the participants were active or former smokers. The mean observed retention time between BL and the 6-month examination was 5.9±0.6 months in the test group and 5.5±0.6 months in the control group. Between the BL and 12-month examination, a mean period of 11.6±0.9 months and 11.8±1.1 months passed for the experimental and control groups, respectively. Significantly more complications (retainer fractures) occurred in the control group (control group: *n* = 7, test group: *n* = 0; *p* < 0.001). Orthodontic treatment characteristics before and after intervention are shown in Table 1.

**Table 1:** Patient characteristics and orthodontic treatment characteristics

	Test (fixed retainer)		Control (removable retainer)	
	N/% or Mean±SD	Median (IQR)	N/% or Mean±SD	Median (IQR)
Gender				
Female	11 (73.3)		11 (64.7)	
Male	4 (26.7)		6 (35.3)	
Age (years)	16.1±4.2	15.0 (14.0/17.0)	17.1±5.4	15.0 (14.0/20.0)
Active/former smoker	0 (0)		0 (0)	
Reexamination periods (months)				
BL – 6 months	5.9±0.6	6.0 (5.0/6.0)	5.5±0.6	6.0 (5.0/6.0)
BL – 12 months	11.6±0.9	11.0 (11.0/12.0)	11.8±1.1	12.0 (11.0/12.0)
Orthodontic treatment				
Duration orthodontic treatment (months)	19.5±7.7	18.0 (16.0/24.0)	29.6±15.9	26.0 (17.0/38.0)
Inclination of lower incisors before therapy (°)	83.1±9.2	86.4 (75.4/90.5)	78.1±20.9	80.0 (77.8/90.4)
Inclination of lower incisors after therapy (°)	80.9±6.3	80.4 (74.6/86.5)	74.7±19.3	79.3 (72.5/85.6)
Inclination of lower incisors change (°)	-2.3±7.1	-4.4 (-6.6/4.3)	-3.4±3.4	-3.7 (-5.5/-1.2)
Intercanine-distance before therapy (mm)	26.5±1.4	26.6 (25.4/26.8)	25.9±1.9	25.9 (24.4/27.4)
Intercanine-distance after therapy (mm)	26.0±1.3	26.0 (24.7/26.8)	26.4±1.2	26.0 (25.5/27.3)
Intercanine-distance change (mm)	-0.5±1.3	-0.7 (-1.3/0.6)	0.5±1.5	0.4 (-0.3/1.4)
Interproximal reduction [IPR]	13 (86.7)^1^		9 (52.9)^1^	
Dental space deficiency	9 (60.0)		13 (76.5)	
Mean fixed retainer distance to CEJ (mm)	5.2±0.5	5.0 (4.8/5.7)	–	–
Complications (retainer fracture/debonding of the retainer)	0 (0.0)^1^	–	7 (41.2)^1^	

SD = standard deviation, IQR = interquartile range, BL = baseline, CEJ = cementoenamel junction

^1^ in-between group comparisons (Mann-Whitney-U-Test or Fisher’s exact test < 0.05)

### Clinical parameters

Mean REC, PPD and CAL values for the BL, 6- and 12-month examinations for both groups are presented in Tables 2, 3 and 4. Both groups demonstrated mean RECs of < 0.5 mm, but they differed significantly after 6 months (test group: 0.02±0.03 mm; control group: 0.00±0.00 mm; *p* = 0.022). There were no significant intra-group differences (Table 2). Differences in mean REC values between BL and 6-month measurements were significantly lower in the test group compared to the control group (*p* < 0.001). The same was true for lingual and buccal measurements and for the differences between BL and 12-month measurements (*p* < 0.001). Mean PPD decreased between the BL and 12-month measurements in both groups (test group BL: 2.05±0.50 mm, 12 months: 1.80±0.33 mm; control group BL: 1.92±0.31 mm, 12 months: 1.76±0.21 mm). The inter-group and intra-group pairwise comparisons were not significant (Table 3). CAL increased slightly in the test group between the BL and 12-month measurement (baseline: 0.35±0.93 mm; 12 months: 0.44±0.93 mm), while it decreased slightly in the control group (baseline: 0.41±0.92 mm, 12 months: 0.20±0.58 mm). No significant inter- and intra-group differences were found either (Table 4). The subgroup analysis of buccal PPD and CAL values revealed significant changes between the BL and 6-month measurements (PPD: *p* = 0.018, CAL: *p* = 0.014) and BL and 12-month measurements (PPD: *p* = 0.043, CAL: *p* = 0.042). Subgroup analysis of lingual sites did not yield additional results (Tables 2, 3 and 4).

**Table 2:** Gingival recessions (mm) at baseline, 6 and 12 months after orthodontic treatment

		Test (fixed retainer)			Control (removable retainer)		
		REC	REC (buccal)	REC (lingual)	REC	REC (buccal)	REC (lingual)
Baseline	Mean±SD	0.01±0.02	0.00±0.02	0.01±0.03	0.02±0.05	0.03±0.10	0.00±0.00
	Median (IQR)	0.0 (0.0/0.0)	0.0 (0.0/0.0)	0.0 (0.0/0.0)	0.0 (0.0/0.0)	0.0 (0.0/0.0)	0.0 (0.0/0.0)
6 months	Mean±SD	0.02±0.03^1^	0.03±0.05^1^	0.00±0.00	0.00±0.00^1^	0.00±0.00^1^	0.00±0.00
	Median (IQR)	0.0 (0.0/0.0)	0.0 (0.0/0.0)	0.0 (0.0/0.0)	0.0 (0.0/0.0)	0.0 (0.0/0.0)	0.0 (0.0/0.0)
12 months	Mean±SD	0.04±0.13	0.03±0.10	0.04±0.16	0.01±0.03	0.02±0.06	0.00±0.00
	Median (IQR)	0.0 (0.0/0.0)	0.0 (0.0/0.0)	0.0 (0.0/0.0)	0.0 (0.0/0.0)	0.0 (0.0/0.0)	0.0 (0.0/0.0)

SD = standard deviation, IQR = interquartile range, REC = recession

^A,B,C^ intra-group comparison (Friedman-Test < 0.05; A: Baseline – 6 months, B: 6–12 months, C: Baseline – 12 months)

^1^ inter-group comparisons (Mann-Whitney-U-Test < 0.05)

**Table 3:** Periodontal probing depths (mm) at baseline, 6 and 12 months after orthodontic treatment

		Test (fixed retainer)			Control (removable retainer)		
		PPD	PPD (buccal)	PPD (lingual)	PPD	PPD (buccal)	PPD (lingual)
Baseline	Mean±SD	2.05±0.50	2.14±0.53^A,C^	1.97±0.52	1.92±0.31	2.02±0.40	1.83±0.32
	Median (IQR)	2.08 (1.67/2.39)	2.22 (1.67/2.61)	1.89 (1.56/2.33)	1.89 (1.67/2.11)	2.17 (1.73/2.36)	1.78 (1.56/2.06)
6 months	Mean±SD	1.78±0.27	1.80±0.33^A^	1.76±0.27	1.74±0.24	1.84±0.32	1.63±0.19
	Median (IQR)	1.69 (1.61/2.06)	1.78 (1.67/2.06)	1.72 (1.56/1.83)	1.67 (1.52/1.96)	1.67 (1.61/2.14)	1.67 (1.50/1.72)
12 months	Mean±SD	1.80±0.33	1.83±0.35^C^	1.76±0.35	1.76±0.21	1.79±0.29	1.73±0.22
	Median (IQR)	1.81 (1.67/2.08)	1.72 (1.67/2.11)	1.89 (1.67/1.94)	1.72 (1.63/1.92)	1.67 (1.59/1.94)	1.72 (1.61/1.81)

SD = standard deviation, IQR = interquartile range, PPD = periodontal probing depth

^A,B,C^ intra-group comparison (repeated measures ANOVA < 0.05; A: Baseline – 6 months, B: 6–12 months, C: Baseline – 12 months)

^1^ inter-group comparisons (t-test < 0.05)

**Table 4:** Clinical attachment level (mm) at baseline, 6 and 12 months after orthodontic treatment

		Test (fixed retainer)			Control (removable retainer)		
		CAL	CAL (buccal)	CAL (lingual)	CAL	CAL (buccal)	CAL (lingual)
Baseline	Mean±SD	0.35±0.93	0.15±0.59	0.65±0.80	0.41±0.92	0.44±1.0	0.00±0.00
	Median (IQR)	0.00 (0.00/0.00)	0.00 (0.00/0.00)	0.00 (0.00/0.00)	0.00 (0.00/0.00)	0.00 (0.00/0.00)	0.00 (0.00/0.00)
6 months	Mean±SD	0.64±0.96	0.69±1.01	0.00±0.00	0.00±0.00	0.00±0.00	0.00±0.00
	Median (IQR)	0.00 (0.00/1.81)	0.00 (0.00/1.94)	0.00 (0.00/0.00)	0.00 (0.00/0.00)	0.00 (0.00/0.00)	0.00 (0.00/0.00)
12 months	Mean±SD	0.44±0.93	0.46±0.95	0.18±0.69	0.20±0.58	0.21±0.59	0.00±0.00
	Median (IQR)	0.00 (0.00/0.00)	0.00 (0.00/0.00)	0.00 (0.00/0.00)	0.00 (0.00/0.00)	0.00 (0.00/0.00)	0.00 (0.00/0.00)

SD = standard deviation, IQR = interquartile range, CAL = clinical attachment level

^A,B,C^ intra-group comparison (repeated measures ANOVA < 0.05; A: Baseline – 6 months, B: 6–12 months, C: Baseline – 12 months)

^1^ inter-group comparisons (t-test < 0.05)

Table 5 demonstrates the absolute and relative distributions of the dichotomised parameters: PlI, GI, and BOP. In both groups, the number of participants with a PlI ≥ 1 increased over time, while GI and BOP ≥ 1 decreased. PlI ≥ 1 occurred significantly more frequently in the control group at BL and 6-month measurements. However, this difference disappeared at the 12-month measurement. At BL there were significantly more participants with a PlI ≥ 1 in the test group when all measured sites (*p* = 0.038) were considered. This was also the case after 6 months (*p* = 0.042). Furthermore, significantly more participants in the test group had a PlI ≥ 1 in the subgroup analysis after the 6-month (lingual; *p* = 0.030) measurements. Within the control group, there was a significant increase in participants with a PlI ≥ 1 between BL and 12-month examinations (*p* = 0.028) and a significant decrease in participants with a GI ≥ 1 between the BL and 6-month examinations (*p* = 0.020). The latter measurements also showed significant differences in the subgroup analysis (lingual and buccal: *p* = 0.001) (Table 5).

**Table 5:** Plaque-Index (dichotomous), Gingival-Index (dichotomous) and Bleeding on probing at baseline, 6 and 12 months after orthodontic treatment

		Test (fixed retainer)			Control (removable retainer)		
		PlI	PlI (buccal)	PlI (lingual)	PlI	PlI (buccal)	PlI (lingual)
Baseline	N	4	3	1	0^C^	0	0
	%	26.7	20.0	6.7	0	0	0
6 months	N	8^1^	5	5^1^	2^1^	2	0^1^
	%	53.4	33.4	33.4	11.8	11.8	0
12 months	N	9	9	5	6^C^	4	3
	%	60.0	60.0	33.4	35.3	23.6	17.8
		GI	GI (buccal)	GI (lingual)	GI	GI (buccal)	GI (lingual)
Baseline	N	13	12	11	14^A^	14^A^	9^A,C^
	%	86.7	80.0	73.3	82.3	82.3	53.0
6 months	N	6	5	3	6^A^	3^A^	4^A^
	%	40.0	33.3	20.0	35.2	17.8	23.6
12 months	N	6	6	2	8	8	3^C^
	%	40.0	40.0	13.4	47.0	47.0	17.8
		BOP	BOP (buccal)	BOP (lingual)	BOP	BOP (buccal)	BOP (lingual)
Baseline	N	10^A^	7	7^C^	11	5	5
	%	66.7	46.7	46.7	64.7	29.4	29.4
6 months	N	4^A^	4	3	6	4	3
	%	26.7	26.7	20.0	35.2	23.5	27.6
12 months	N	7	3	5^C^	8	7	1
	%	46.7	20.0	33.3	47.1	41.2	5.9

PlI = plaque index, GI = gingival index, BOP = bleeding on probing

^A,B,C^ intra-group comparison (Cochran’s test < 0.05; A: Baseline – 6 months, B: 6–12 months, C: Baseline – 12 months)

^1^ inter-group comparisons (chi^2^-test or Fisher’s exact test < 0.05)

### Gingival Health

The distribution of GH among both groups and the examination time points is depicted in Table 6. While the number of participants in the test group demonstrating GH remained almost unchanged over the examination period (BL: *n* = 10/66.7%, 6 months: *n* = 9/60.0%, 12 months: *n* = 11/73.3%), it increased in the control group (BL: *n* = 10/58.8%, 6 months: *n* = 16/94.1%, 12 months: *n* = 15/88.2%). The increase from BL to 6 months was significant (*p* = 0.043). Inter-group comparisons revealed no significant difference in the number of participants demonstrating GH.

**Table 6:** Distribution of gingival health among groups and re-examination time points

		Gingival health		
		Test (fixed retainer)	Control (removable retainer)	*p*-value
Baseline	N	10	10	1.000
	%	66.7	58.8	
6 months	N	9	16	0.066
	%	60.0	94.1	
12 months	N	11	15	0.766
	%	73.3	88.2	
*p*-value	BL – 6 months	0.687	0.043	
	6 months – 12 months	0.687	1.000	
	BL – 12 months	0.687	0.124	

BL = baseline

## Discussion

The present study aimed to evaluate the influence of fixed orthodontic retainers on GH and REC of mandibular anterior teeth. Inter-group comparisons failed to show significant differences in the number of participants demonstrating GH. Intra-group comparisons revealed a significant increase in the number of participants demonstrating GH in the control group between the BL and 6-month examinations. There was no significant change in the number of participants demonstrating GH between the BL and 12-month examination, both inter- and intra-group. No significant differences in REC values were observed.

Rody et al. investigated the effects of different orthodontic retention protocols on periodontal health by assessing molecular markers in the gingival crevicular fluid of mandibular incisors [12]. They concluded that the use of fixed retainers did not significantly affect the parameters of periodontal health. However, the present study found that in both groups, the number of participants with a PlI ≥ 1 increased over time. Other previous studies that investigated the influence of fixed retainers on the health of the surrounding periodontal tissues have also demonstrated that fixed retainers tend to increase plaque accumulation [8, 13, 14, 17, 40]. However, this could be due to the clinically evident plaque-retentive characteristic of fixed retainers and the effect these retainers have on oral hygiene performance [8, 41, 42].

In the present study, the increase in participants demonstrating GH in the control group after 6 months could possibly be explained by the fact that these participants could reach all their teeth and proximal surfaces without difficulty, whereas the test group had to adapt to a more difficult cleaning situation. It has already been reported that vacuum-formed removable retainers seem to not increase plaque accumulation [16, 43]. To test the effect of fixed retainers on GH in the present study, the authors modified the vacuum-formed retainer to enable similar plaque accumulation.

In the present study, mean PPD decreased between the BL and 12-month examinations in both groups. In a series of studies, Artun et al. investigated the periodontal health effects of fixed and removable retainers, with a maximum follow-up period of 4 years [9, 44, 45]. In these studies, the definition of periodontal health was based on the Periodontal Disease Index system [46] and GI [47]. Similar to the findings in the current study, Artun et al. reported less gingival bleeding over the observed period. They also confirmed the plaque-promoting effect of bonded retainers, finding that they caused no apparent damage to the periodontal tissues and did not have a negative impact on periodontal health maintenance [44].

In contrast to the findings of the current study, as well as some of the previously mentioned studies, Pandis et al. reported an increase in PPD, greater calculus accumulation, and greater marginal recession in 32 patients with mandibular fixed (stainless-steel wire) retainers that were observed, on average, for 9.7 years [15]. Levin et al. reported an increased incidence of REC and BOP in association with orthodontic treatment and fixed retainers. The study involved 92 post-orthodontic patients, who were categorised by the presence or absence of fixed retainers. However, that study did not have a defined follow-up period, and their observations resulted from the comparison between groups after an arbitrary post-orthodontic therapy period [14].

As previously mentioned, in the current study, there was a significant increase in GH at the 6-month examination in the control group. In this context it is important to clarify the terminology of ‘GH’ or ‘periodontal health’ within the context of orthodontic studies. To the best of the authors’ knowledge, the definition of GH used in the present study, which was based on the 2018 classification of periodontal diseases and conditions, has not been used in comparable studies [7]. Much of the previous orthodontic literature reports on GH or periodontal health based on different single clinical parameters, such as low GI, PlI or PPD values. Therefore, there is limited comparability between the current study and previous studies.

In the control group, more participants demonstrated GH after 12 months. However, these participants had to be more compliant and 41.2% of these participants experienced a retainer fracture. No complications were registered among the test group participants during the examination period. However, it is important to note that the vacuum-formed retainers used in this study were modified to closely simulate the feeling of not having a retainer. This reduction in material inevitably led to a predetermined fracture point. Hawley retainers could have been used in the control group, which would have likely reduced the complication rate.

A recent randomised clinical trial compared two fixed retainers (3-strand round twisted and 8-strand rectangular braided fixed) over 2 years and demonstrated an overall risk for first-time failure of 52.3% [48]. Another study reported a total detachment rate of 22.54% for a multistrand stainless steel wire retainer and 14.45% for polyethylene ribbon-reinforced resin retainer after a follow-up period of 12 months [49]. Bolla et al. compared the bond failure and fracture rates of two types of bonded lingual orthodontic retainers (glass fibre-reinforced [GFR] and multistrand stainless steel wire) across a 6-year retention period, finding similar failure and fracture rates across both types. They reported a mandibular fracture rate of 8.82% for the GFR group and 15.62% for the multistrand wire group [50]. The present study did not observe any complications with the fixed retainers over the 12-month examination period. This is probably due to the young age of the study cohort and their initially healthy periodontal conditions [51].

Regarding the potential relationship between the occurrence of REC and the use of orthodontic retainers, the present study found a mean recession of < 0.5 mm in both groups. Although REC appeared more pronounced in the test group, no clinically relevant impact on the progression or development of REC was observed within 12 months. REC occurs more frequently with increasing age, which is one possible explanation as to why it was not often observed in the current study cohort [52]. However, a link between orthodontic movement in different age groups and REC has not been reported in previous studies [53, 54]. A recent review stated that more proclined teeth and teeth that have moved out of the osseous envelope of the alveolar process may have a higher tendency to develop REC [18]. However, the studies included in the review had high degrees of variety in their treatment duration, applied forces, control groups, and degree of movement. Only one study was able to provide a precise statement on the link between orthodontic movement and REC, reporting that a final lower incisor inclination of more than 95° in relation to the mandibular plane was directly related to more frequent and severe REC in the mandibular central incisors. Furthermore, the amount of proclination was not important; only the final inclination impacted REC [55]. The mean distance of 5 mm between the gingival margin and the retainer in the present study could also be responsible for the inconspicuous periodontal parameters in the test group. A comparable study found greater recessions at a smaller distance (≤ 1.25 mm) than with retainers bonded further incisally [14].

Determining whether REC is caused by orthodontic treatment or the retainer remains difficult, as REC occurring during retention could be a delayed effect of the orthodontic movement. Additionally, due to the more complex aetiology of REC, there may be other factors that impact its occurrence besides orthodontic extent, movement direction, and post-orthodontic retention. In particular, the gingival phenotype is associated with an increased risk of developing REC [56, 57]. The current study did not assess this parameter, which is a limitation.

Juloski et al. concluded that the prevalence of REC in patients 5 years after orthodontic treatment, with or without retainers, was similar to the prevalence in untreated individuals of the same age [58]. However, another study that assessed the incidence of REC over 10 years found that females reported increasing REC and felt that their teeth were getting longer [59]. Another recent study found that having a mandibular retainer was associated with decreased overall treatment satisfaction [59]. Participant-reported outcomes were not assessed in the current study.

This study has two fundamental limitations. On the one hand, there is a lack of data on the orthodontic pre-treatment situations in order to estimate how pronounced the movements of the lower anterior incisors that occurred as part of the orthodontic therapy were. On the other hand, a longer follow-up period might have shown clearer differences between both groups.

As practical implication of this clinical trial the choice of a fixed or removable retainer does not have to be dependent on GH that can be achieved in the short-term. Here, other parameters such as the stability of the orthodontic result and the patient’s compliance when wearing the retainer should be given priority.

Future studies should consider establishing comparability between participant groups in age, gingival phenotype, orthodontic treatment scope, participant-reported outcome measures, and the type of post-orthodontic retention.

## Conclusion

Overall, 73.3% of young orthodontically treated patients with fixed steel retainers demonstrated GH 12 months after the intervention. This proportion was comparable to the control group (88.2%). There were no significant inter- and intra-group differences in REC values observed throughout the study period.

## Data Availability

The data of this study are available from the corresponding author upon reasonable request.
